# (2*S*,3*S*)-3-(3-Bromo­phen­yl)-6,6-dimethyl-2-nitro-2,3,6,7-tetra­hydro­benzo­furan-4(5*H*)-one

**DOI:** 10.1107/S160053681301920X

**Published:** 2013-07-17

**Authors:** Yifeng Wang, Minjie Yao, Kun Dong, Danqian Xu

**Affiliations:** a Catalytic Hydrogenation Research Center, Zhejiang University of Technology, Hangzhou 310014, People’s Republic of China

## Abstract

The title compound, C_16_H_16_BrNO_4_, has two adjacent chiral C atoms and both have an *S* configuration. The fused cyclo­hex-2-enone and di­hydro­furan rings both adopt envelope conformations, with the quaternary C atom and the nitro-substituted C atoms as the respective flap. The flap atoms lie 0.607 (3) and −0.253 (2) Å, respectively, from the mean plane of the remaining ring atoms on opposite sides. The dihedral angle between the mean plane of the four coplanar atoms of the di­hydro­furan ring and the phenyl ring is 86.16 (3)°. In the crystal, mol­ecules are linked by weak C—H⋯O inter­actions, forming a ladder motif parallel to the *b* axis.

## Related literature

For the occurrence of di­hydro­furans in nature and their synthetic applications, see: Fraga (1992 ▶); Lipshutz (1986 ▶). For synthetic procedures, see: Fan *et al.* (2010 ▶); Rueping *et al.* (2010 ▶).
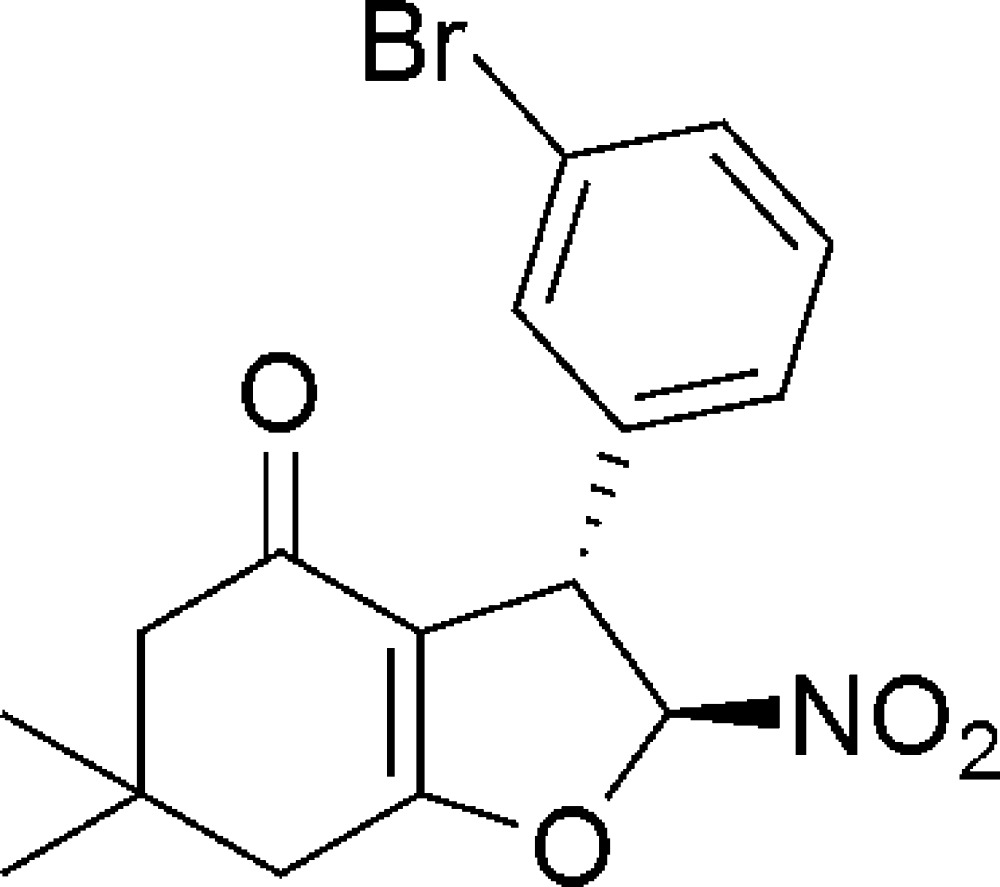


## Experimental

### 

#### Crystal data

C_16_H_16_BrNO_4_
*M* *_r_* = 366.21
Orthorhombic, 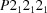

*a* = 6.6799 (7) Å
*b* = 7.3713 (9) Å
*c* = 32.9075 (14) Å
*V* = 1620.4 (3) Å^3^
*Z* = 4
Mo *K*α radiation
μ = 2.55 mm^−1^
*T* = 296 K
0.38 × 0.36 × 0.31 mm

#### Data collection

Rigaku R-AXIS RAPID diffractometer
Absorption correction: multi-scan (*ABSCOR*; Higashi, 1995 ▶) *T*_min_ = 0.384, *T*_max_ = 0.455
13344 measured reflections
2997 independent reflections
1857 reflections with *I* > 2σ(*I*)
*R*_int_ = 0.066

#### Refinement

*R*[*F* ^2^ > 2σ(*F* ^2^)] = 0.051
*wR*(*F* ^2^) = 0.108
*S* = 1.00
2997 reflections
200 parameters
H-atom parameters constrained
Δρ_max_ = 0.51 e Å^−3^
Δρ_min_ = −0.46 e Å^−3^
Absolute structure: Flack (1983 ▶), 1221 Friedel pairs
Absolute structure parameter: 0.003 (18)

### 

Data collection: *PROCESS-AUTO* (Rigaku, 2006 ▶); cell refinement: *PROCESS-AUTO*; data reduction: *CrystalStructure* (Rigaku, 2007 ▶); program(s) used to solve structure: *SHELXS97* (Sheldrick, 2008 ▶); program(s) used to refine structure: *SHELXL97* (Sheldrick, 2008 ▶); molecular graphics: *ORTEP-3 for Windows* (Farrugia, 2012 ▶); software used to prepare material for publication: *WinGX* (Farrugia, 2012 ▶).

## Supplementary Material

Crystal structure: contains datablock(s) General, I. DOI: 10.1107/S160053681301920X/fy2101sup1.cif

Structure factors: contains datablock(s) I. DOI: 10.1107/S160053681301920X/fy2101Isup2.hkl

Supplementary material file. DOI: 10.1107/S160053681301920X/fy2101Isup3.cml

Additional supplementary materials:  crystallographic information; 3D view; checkCIF report

## Figures and Tables

**Table 1:** Hydrogen-bond geometry (Å, °)

*D*—H⋯*A*	*D*—H	H⋯*A*	*D*⋯*A*	*D*—H⋯*A*
C1—H1⋯O1^i^	0.98	2.29	3.165 (6)	148
C5—H5*A*⋯O2^ii^	0.97	2.66	3.351 (6)	129
C14—H14⋯O1^iii^	0.93	2.65	3.559 (7)	167

Symmetry codes: (i) 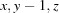; (ii) 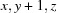; (iii) 
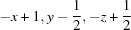
.

**Fig. 1.**
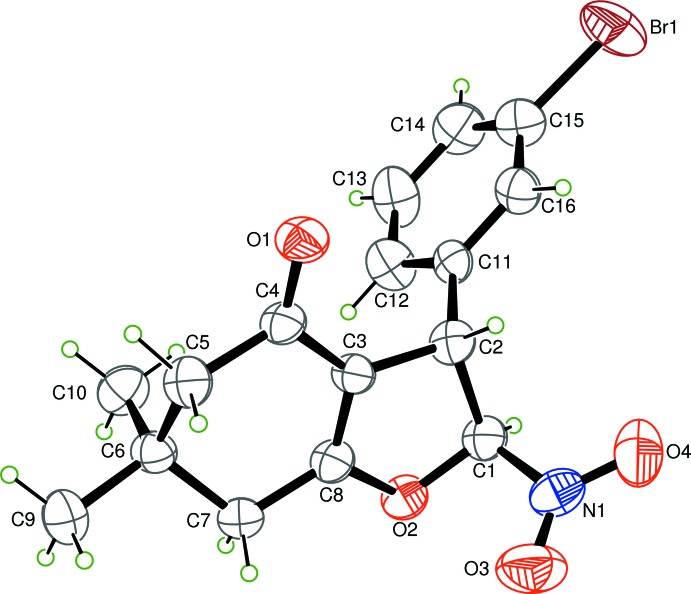
The asymmetric unit of the structure of the title compound. Displacement ellipsoids are drawn at the 50% probability level.

**Fig. 2.**
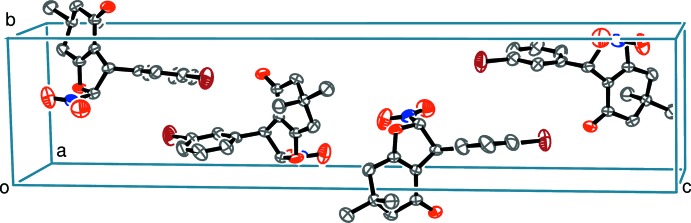
The molecular packing of the title compounds.

## supplementary crystallographic information

### Comment

Dihdrofurans are found in many naturally occurring compounds and are used as versatile intermediates in organic and natural product synthesis (Fraga, 1992; Lipshutz, 1986). Organocatalytic asymmetric domino reactions have received increasing attention in the synthetic community recently. The title compound, which was readily synthesized by the organocatalytic Michael-S_N_2 reaction of 5,5-dimethylcyclohexane-1,3-dione to (*E*)-1-bromo-3-(2-bromo-2-nitrovinyl)benzene (Fan *et al.*, 2010; Rueping *et al.*, 2010), could act as an intermediate in organic and natural product synthesis. In this article, the crystal structure of the title compoud (2*S*,3*S*)-3-(3-bromophenyl)-6,6-dimethyl-2- nitro-2,3,6,7-tetrahydrobenzofuran-4(5*H*)-one is described (Fig. 1).

### Experimental

To a solution of 5,5-dimethylcyclohexane-1,3-dione (1.2 mmol) and (*E*)-1-bromo-3-(2-bromo-2-nitrovinyl)benzene (1 mmol) in CHCl_3_ (3 ml) was added 1-(3,5-bis(trifluoromethyl)phenyl)-3-((*S*) -(6-methoxyquinolin-4-yl)((2*S*,4*S*,8*R*)-8-vinylquinuclidin-2 -yl)methyl)thiourea (0.025 mmol) as catalyst and *N*,*N*-diisopropylethylamine (DIPEA, 0.3 mmol) as the base. The mixture was stirred at room temperature for 12 h (monitored by TLC). Then the solvent was distilled under vacuum, and the residue was purified by flash column chromatography (silica gel, Hex/AcOEt, *v*/*v*, 3:1) giving the title compound. Single crystals were obtained by slow evaporation of a CH_2_Cl_2_ and *i*PrOH solution (*v*/*v*, 1:1).

### Refinement

Methyl H atoms were placed in calculated positions with C—H = 0.96 (1) Å and the methyl torsion was refined to fit the electron density with *U*_iso_(H) = 1.5*U*_eq_(C). Other H atoms were placed in calculated positions with C—H = 0.98 (1) Å (CH), C—H = 0.97 (1) Å (CH_2_), C—H = 0.93 Å (aromatic). All H atoms included in the final cycles of refinement using a riding model, with *U*_iso_(H) = 1.2*U*_eq_(C).

### Crystal data

**Table:**

C_16_H_16_BrNO_4_	*F*(000) = 744
*M**_r_* = 366.21	*D*_x_ = 1.501 Mg m^−^^3^
Orthorhombic, *P*2_1_2_1_2_1_	Mo *K*α radiation, λ = 0.71073 Å
Hall symbol: P 2ac 2ab	Cell parameters from 8549 reflections
*a* = 6.6799 (7) Å	θ = 3.0–27.4°
*b* = 7.3713 (9) Å	µ = 2.55 mm^−^^1^
*c* = 32.9075 (14) Å	*T* = 296 K
*V* = 1620.4 (3) Å^3^	Chunk, colourless
*Z* = 4	0.38 × 0.36 × 0.31 mm

### Data collection

**Table:**

Rigaku R-AXIS RAPID diffractometer	2997 independent reflections
Radiation source: rotating anode	1857 reflections with *I* > 2σ(*I*)
Graphite monochromator	*R*_int_ = 0.066
Detector resolution: 10.00 pixels mm^-1^	θ_max_ = 25.5°, θ_min_ = 3.0°
ω scans	*h* = −8→8
Absorption correction: multi-scan (*ABSCOR*; Higashi, 1995)	*k* = −8→8
*T*_min_ = 0.384, *T*_max_ = 0.455	*l* = −39→39
13344 measured reflections	

### Refinement

**Table:**

Refinement on *F*^2^	Hydrogen site location: inferred from neighbouring sites
Least-squares matrix: full	H-atom parameters constrained
*R*[*F*^2^ > 2σ(*F*^2^)] = 0.051	*w* = 1/[σ^2^(*F*_o_^2^) + (0.0122*P*)^2^ + 2.5684*P*] where *P* = (*F*_o_^2^ + 2*F*_c_^2^)/3
*wR*(*F*^2^) = 0.108	(Δ/σ)_max_ < 0.001
*S* = 1.00	Δρ_max_ = 0.51 e Å^−^^3^
2997 reflections	Δρ_min_ = −0.46 e Å^−^^3^
200 parameters	Extinction correction: *SHELXL97* (Sheldrick, 2008), Fc^*^=kFc[1+0.001xFc^2^λ^3^/sin(2θ)]^-1/4^
0 restraints	Extinction coefficient: 0.0068 (8)
Primary atom site location: structure-invariant direct methods	Absolute structure: Flack (1983), 1221 Friedel pairs
Secondary atom site location: difference Fourier map	Flack parameter: 0.003 (18)

### Special details

**Table:**

Geometry. All e.s.d.'s (except the e.s.d. in the dihedral angle between two l.s. planes) are estimated using the full covariance matrix. The cell e.s.d.'s are taken into account individually in the estimation of e.s.d.'s in distances, angles and torsion angles; correlations between e.s.d.'s in cell parameters are only used when they are defined by crystal symmetry. An approximate (isotropic) treatment of cell e.s.d.'s is used for estimating e.s.d.'s involving l.s. planes.
Refinement. Refinement of *F*^2^ against ALL reflections. The weighted *R*-factor *wR* and goodness of fit *S* are based on *F*^2^, conventional *R*-factors *R* are based on *F*, with *F* set to zero for negative *F*^2^. The threshold expression of *F*^2^ > σ(*F*^2^) is used only for calculating *R*-factors(gt) *etc*. and is not relevant to the choice of reflections for refinement. *R*-factors based on *F*^2^ are statistically about twice as large as those based on *F*, and *R*- factors based on ALL data will be even larger.

### Fractional atomic coordinates and isotropic or equivalent isotropic displacement parameters (Å^2^)

**Table:**

	*x*	*y*	*z*	*U*_iso_*/*U*_eq_	
C1	0.7480 (8)	0.0956 (7)	0.38377 (16)	0.0479 (13)	
H1	0.7209	−0.0091	0.3663	0.058*	
C2	0.7540 (7)	0.2708 (7)	0.35851 (13)	0.0445 (12)	
H2	0.8923	0.3142	0.3564	0.053*	
C3	0.6377 (8)	0.3915 (6)	0.38649 (13)	0.0407 (12)	
C4	0.6168 (8)	0.5877 (7)	0.38557 (14)	0.0447 (12)	
C5	0.5014 (8)	0.6680 (7)	0.42097 (15)	0.0518 (14)	
H5A	0.4417	0.7813	0.4122	0.062*	
H5B	0.5956	0.6963	0.4425	0.062*	
C6	0.3350 (8)	0.5471 (7)	0.43873 (14)	0.0457 (13)	
C7	0.4211 (8)	0.3581 (7)	0.44910 (14)	0.0510 (14)	
H7A	0.4962	0.3648	0.4743	0.061*	
H7B	0.3123	0.2726	0.4529	0.061*	
C8	0.5546 (7)	0.2937 (7)	0.41577 (13)	0.0441 (12)	
C9	0.2524 (9)	0.6355 (8)	0.47723 (16)	0.0662 (17)	
H9A	0.3577	0.6471	0.4969	0.099*	
H9B	0.1473	0.5614	0.4882	0.099*	
H9C	0.2004	0.7534	0.4708	0.099*	
C10	0.1679 (8)	0.5273 (8)	0.40747 (16)	0.0604 (16)	
H10A	0.2203	0.4722	0.3833	0.091*	
H10B	0.1147	0.6448	0.4010	0.091*	
H10C	0.0635	0.4524	0.4184	0.091*	
C11	0.6670 (6)	0.2454 (7)	0.31637 (13)	0.0424 (12)	
C12	0.4643 (8)	0.2067 (8)	0.31090 (16)	0.0625 (16)	
H12	0.3787	0.1999	0.3331	0.075*	
C13	0.3924 (9)	0.1789 (9)	0.27221 (18)	0.0753 (19)	
H13	0.2568	0.1561	0.2684	0.090*	
C14	0.5172 (10)	0.1840 (9)	0.23926 (17)	0.0712 (19)	
H14	0.4667	0.1635	0.2133	0.085*	
C15	0.7173 (8)	0.2196 (9)	0.24461 (15)	0.0589 (15)	
C16	0.7912 (8)	0.2488 (8)	0.28321 (13)	0.0526 (13)	
H16	0.9272	0.2710	0.2868	0.063*	
N1	0.9468 (8)	0.0706 (7)	0.40626 (16)	0.0636 (13)	
O1	0.6964 (5)	0.6824 (5)	0.35982 (10)	0.0568 (10)	
O2	0.5974 (6)	0.1130 (4)	0.41360 (10)	0.0513 (9)	
O3	0.9540 (7)	0.0943 (7)	0.44265 (13)	0.0948 (16)	
O4	1.0920 (7)	0.0381 (7)	0.38474 (15)	0.0926 (15)	
Br1	0.89492 (12)	0.22108 (12)	0.200225 (18)	0.1030 (4)	

### Atomic displacement parameters (Å^2^)

**Table:**

	*U*^11^	*U*^22^	*U*^33^	*U*^12^	*U*^13^	*U*^23^
C1	0.058 (3)	0.034 (3)	0.052 (3)	0.000 (3)	0.001 (3)	−0.005 (3)
C2	0.053 (3)	0.032 (3)	0.048 (2)	−0.006 (3)	−0.001 (2)	−0.004 (3)
C3	0.055 (3)	0.031 (3)	0.036 (2)	−0.003 (3)	0.004 (2)	−0.003 (2)
C4	0.057 (3)	0.032 (3)	0.045 (3)	−0.001 (3)	0.000 (3)	−0.001 (2)
C5	0.064 (3)	0.036 (3)	0.055 (3)	−0.003 (3)	0.001 (3)	−0.011 (2)
C6	0.056 (3)	0.042 (3)	0.039 (3)	−0.001 (3)	0.002 (2)	−0.002 (2)
C7	0.056 (3)	0.051 (4)	0.047 (3)	0.003 (3)	0.005 (3)	0.007 (2)
C8	0.056 (3)	0.029 (3)	0.048 (3)	−0.002 (3)	0.002 (2)	0.000 (2)
C9	0.073 (4)	0.063 (4)	0.062 (3)	0.011 (3)	0.015 (3)	−0.010 (3)
C10	0.060 (4)	0.061 (4)	0.060 (3)	−0.002 (3)	−0.013 (3)	−0.001 (3)
C11	0.049 (3)	0.036 (3)	0.042 (2)	0.001 (3)	0.002 (2)	−0.009 (2)
C12	0.056 (3)	0.073 (4)	0.058 (3)	−0.010 (3)	−0.002 (3)	−0.013 (3)
C13	0.058 (4)	0.091 (5)	0.077 (4)	−0.001 (4)	−0.005 (3)	−0.029 (4)
C14	0.089 (5)	0.072 (5)	0.053 (3)	0.006 (4)	−0.015 (3)	−0.018 (3)
C15	0.068 (4)	0.058 (4)	0.051 (3)	−0.004 (3)	−0.003 (3)	−0.007 (3)
C16	0.056 (3)	0.051 (4)	0.050 (3)	0.002 (3)	−0.003 (2)	−0.007 (3)
N1	0.076 (4)	0.047 (3)	0.068 (3)	−0.002 (3)	−0.009 (3)	0.008 (3)
O1	0.076 (2)	0.036 (2)	0.058 (2)	−0.0071 (19)	0.0121 (19)	0.0059 (18)
O2	0.065 (2)	0.027 (2)	0.062 (2)	−0.0025 (19)	0.008 (2)	0.0077 (16)
O3	0.084 (3)	0.138 (4)	0.062 (3)	−0.009 (3)	−0.014 (3)	0.024 (3)
O4	0.071 (3)	0.103 (4)	0.104 (3)	0.026 (3)	0.011 (3)	−0.006 (3)
Br1	0.1209 (6)	0.1395 (8)	0.0487 (3)	−0.0071 (6)	0.0208 (4)	−0.0096 (4)

### Geometric parameters (Å, º)

**Table:**

C1—O2	1.411 (6)	C8—O2	1.365 (5)
C1—N1	1.532 (7)	C9—H9A	0.9600
C1—C2	1.536 (7)	C9—H9B	0.9600
C1—H1	0.9800	C9—H9C	0.9600
C2—C3	1.498 (6)	C10—H10A	0.9600
C2—C11	1.515 (6)	C10—H10B	0.9600
C2—H2	0.9800	C10—H10C	0.9600
C3—C8	1.325 (6)	C11—C16	1.371 (6)
C3—C4	1.453 (7)	C11—C12	1.396 (6)
C4—O1	1.220 (5)	C12—C13	1.376 (7)
C4—C5	1.517 (7)	C12—H12	0.9300
C5—C6	1.540 (7)	C13—C14	1.368 (8)
C5—H5A	0.9700	C13—H13	0.9300
C5—H5B	0.9700	C14—C15	1.373 (8)
C6—C10	1.525 (7)	C14—H14	0.9300
C6—C9	1.528 (7)	C15—C16	1.380 (6)
C6—C7	1.545 (7)	C15—Br1	1.882 (5)
C7—C8	1.491 (6)	C16—H16	0.9300
C7—H7A	0.9700	N1—O3	1.211 (6)
C7—H7B	0.9700	N1—O4	1.224 (6)
			
O2—C1—N1	107.0 (4)	C3—C8—C7	127.8 (5)
O2—C1—C2	108.6 (4)	O2—C8—C7	118.3 (4)
N1—C1—C2	109.9 (4)	C6—C9—H9A	109.5
O2—C1—H1	110.4	C6—C9—H9B	109.5
N1—C1—H1	110.4	H9A—C9—H9B	109.5
C2—C1—H1	110.4	C6—C9—H9C	109.5
C3—C2—C11	115.9 (4)	H9A—C9—H9C	109.5
C3—C2—C1	98.8 (4)	H9B—C9—H9C	109.5
C11—C2—C1	112.4 (4)	C6—C10—H10A	109.5
C3—C2—H2	109.7	C6—C10—H10B	109.5
C11—C2—H2	109.7	H10A—C10—H10B	109.5
C1—C2—H2	109.7	C6—C10—H10C	109.5
C8—C3—C4	121.1 (5)	H10A—C10—H10C	109.5
C8—C3—C2	109.9 (4)	H10B—C10—H10C	109.5
C4—C3—C2	129.0 (4)	C16—C11—C12	119.2 (4)
O1—C4—C3	122.8 (5)	C16—C11—C2	119.6 (4)
O1—C4—C5	122.1 (5)	C12—C11—C2	121.0 (4)
C3—C4—C5	114.9 (4)	C13—C12—C11	119.2 (5)
C4—C5—C6	115.6 (4)	C13—C12—H12	120.4
C4—C5—H5A	108.4	C11—C12—H12	120.4
C6—C5—H5A	108.4	C12—C13—C14	121.1 (6)
C4—C5—H5B	108.4	C12—C13—H13	119.4
C6—C5—H5B	108.4	C14—C13—H13	119.4
H5A—C5—H5B	107.4	C13—C14—C15	119.8 (5)
C10—C6—C9	109.6 (4)	C13—C14—H14	120.1
C10—C6—C7	109.6 (4)	C15—C14—H14	120.1
C9—C6—C7	109.6 (4)	C14—C15—C16	119.8 (5)
C10—C6—C5	109.1 (4)	C14—C15—Br1	121.0 (4)
C9—C6—C5	109.2 (4)	C16—C15—Br1	119.2 (4)
C7—C6—C5	109.7 (4)	C11—C16—C15	120.9 (5)
C8—C7—C6	110.3 (4)	C11—C16—H16	119.6
C8—C7—H7A	109.6	C15—C16—H16	119.6
C6—C7—H7A	109.6	O3—N1—O4	124.7 (5)
C8—C7—H7B	109.6	O3—N1—C1	119.6 (5)
C6—C7—H7B	109.6	O4—N1—C1	115.5 (5)
H7A—C7—H7B	108.1	C8—O2—C1	105.9 (4)
C3—C8—O2	113.9 (4)		
			
O2—C1—C2—C3	16.1 (5)	C6—C7—C8—C3	17.4 (7)
N1—C1—C2—C3	−100.7 (5)	C6—C7—C8—O2	−161.3 (4)
O2—C1—C2—C11	−106.8 (4)	C3—C2—C11—C16	137.5 (5)
N1—C1—C2—C11	136.5 (4)	C1—C2—C11—C16	−109.9 (6)
C11—C2—C3—C8	110.0 (5)	C3—C2—C11—C12	−46.8 (7)
C1—C2—C3—C8	−10.3 (5)	C1—C2—C11—C12	65.8 (7)
C11—C2—C3—C4	−72.7 (7)	C16—C11—C12—C13	−2.2 (10)
C1—C2—C3—C4	167.0 (5)	C2—C11—C12—C13	−177.9 (5)
C8—C3—C4—O1	177.6 (5)	C11—C12—C13—C14	1.6 (10)
C2—C3—C4—O1	0.6 (9)	C12—C13—C14—C15	−0.7 (11)
C8—C3—C4—C5	2.2 (7)	C13—C14—C15—C16	0.4 (11)
C2—C3—C4—C5	−174.9 (5)	C13—C14—C15—Br1	178.2 (5)
O1—C4—C5—C6	152.9 (5)	C12—C11—C16—C15	1.9 (9)
C3—C4—C5—C6	−31.6 (7)	C2—C11—C16—C15	177.7 (5)
C4—C5—C6—C10	−67.6 (6)	C14—C15—C16—C11	−1.0 (10)
C4—C5—C6—C9	172.6 (5)	Br1—C15—C16—C11	−178.8 (4)
C4—C5—C6—C7	52.5 (6)	O2—C1—N1—O3	−10.3 (7)
C10—C6—C7—C8	76.6 (5)	C2—C1—N1—O3	107.4 (6)
C9—C6—C7—C8	−163.1 (4)	O2—C1—N1—O4	174.1 (5)
C5—C6—C7—C8	−43.2 (5)	C2—C1—N1—O4	−68.1 (6)
C4—C3—C8—O2	−176.5 (4)	C3—C8—O2—C1	9.9 (6)
C2—C3—C8—O2	1.1 (6)	C7—C8—O2—C1	−171.2 (4)
C4—C3—C8—C7	4.7 (8)	N1—C1—O2—C8	102.2 (4)
C2—C3—C8—C7	−177.7 (4)	C2—C1—O2—C8	−16.4 (5)

### Hydrogen-bond geometry (Å, º)

**Table:**

*D*—H···*A*	*D*—H	H···*A*	*D*···*A*	*D*—H···*A*
C1—H1···O1^i^	0.98	2.29	3.165 (6)	148
C5—H5*A*···O2^ii^	0.97	2.66	3.351 (6)	129
C14—H14···O1^iii^	0.93	2.65	3.559 (7)	167

Symmetry codes: (i) *x*, *y*−1, *z*; (ii) *x*, *y*+1, *z*; (iii) −*x*+1, *y*−1/2, −*z*+1/2.

## References

[bb1] Fan, L. P., Li, P., Li, X. S., Xu, D. C., Ge, M. M., Zhu, W. D. & Xie, J. W. (2010). *J. Org. Chem.* **75**, 8716–8719. 10.1021/jo101935k 21090776

[bb2] Farrugia, L. J. (2012). *J. Appl. Cryst.* **45**, 849–854.

[bb3] Flack, H. D. (1983). *Acta Cryst.* A**39**, 876–881.

[bb4] Fraga, B. M. (1992). *Nat. Prod. Rep.* **9**, 217–241. 10.1039/np9920900217 1436737

[bb5] Higashi, T. (1995). *ABSCOR* Rigaku Corporation, Tokyo, Japan.

[bb6] Lipshutz, B. H. (1986). *Chem. Rev.* **86**, 795–819.

[bb7] Rigaku (2006). PROCESS_AUTO. Rigaku Corporation, Tokyo, Japan.

[bb8] Rigaku (2007). *CrystalStructure.* Rigaku Americas, The Woodlands, Texas, USA.

[bb9] Rueping, M., Parra, A., Uria, U., Besselievre, F. & Merino, E. (2010). *Org. Lett.* **12**, 5680–5683. 10.1021/ol102499r 21090798

[bb10] Sheldrick, G. M. (2008). *Acta Cryst.* A**64**, 112–122. 10.1107/S0108767307043930 18156677

